# Advance Care Planning Engagement and End-of-Life Preference Among Older Chinese Americans

**DOI:** 10.1093/geroni/igaa057.2704

**Published:** 2020-12-16

**Authors:** Yaolin Pei, Wei Zhang, Bei Wu

**Affiliations:** 1 New York University, New York, New York, United States; 2 University of Hawaii at Manoa, Honolulu, Hawaii, United States

## Abstract

The study aimed to examine how immigrant status and family relationships are associated with advanced care planning (ACP) engagement and end-of-life (EOL) preference over burial plan among older Chinese Americans, the largest subgroup of Asian Americans. Logistic regressions were used to analyze data from a survey of 430 older Chinese Americans aged 55 and older living in a Honolulu, Hawai’i. Results show that U.S.-born Chinese Americans were more likely to engage in ACP, including willingness thought of EOL care, and discussion about EOL care, and having preference over burial plan, than the foreign-born Chinese American. Family cohesion was not associated with ACP engagement and EOL preference over burial plan. Family conflict increased the possibility of ACP engagement, indicated by willingness thought of ACP, willingness discussion on ACP, and EOL preference over burial plan. The culturally sensitive educational intervention is needed to increase ACP engagement among older Chinese Americans.

